# Multi-omics Analysis of Gut Microbiota and Metabolites in Rats With Irritable Bowel Syndrome

**DOI:** 10.3389/fcimb.2019.00178

**Published:** 2019-05-29

**Authors:** Si Liu, Chaozeng Si, Yang Yu, Guiping Zhao, Lei Chen, Yu Zhao, Zheng Zhang, Hengcun Li, Yang Chen, Li Min, Shutian Zhang, Shengtao Zhu

**Affiliations:** ^1^Beijing Key Laboratory for Precancerous Lesion of Digestive Disease, Department of Gastroenterology, Beijing Friendship Hospital, National Clinical Research Center for Digestive Disease, Beijing Digestive Disease Center, Capital Medical University, Beijing, China; ^2^Department of Operations and Information Management, China-Japan Friendship Hospital, Beijing, China; ^3^MOE Key Laboratory of Bioinformatics, BNRist, Department of Automation, Bioinformatics Division, Center for Synthetic and Systems Biology, Tsinghua University, Beijing, China

**Keywords:** irritable bowel syndrome (IBS), water avoidance stress (WAS), fecal metabolomics profiling, 16S rRNA gene sequencing, correlation analysis

## Abstract

Irritable bowel syndrome (IBS) is a common gastrointestinal dysfunctional disease. The pathophysiology of IBS is, however, largely unknown. This study aimed to determine whether evaluation of fecal metabolite and microbiota profiles may offer an opportunity to identify a novel pathophysiological target for IBS, and to reveal possible gut microbe–metabolite associations. By using gas chromatography coupled to time-of-flight mass spectrometry (GC-TOFMS) and 16S rRNA gene sequencing, we measured fecal metabolites and microbiota of the control and water avoidance stress (WAS)-induced IBS rats. We found a significantly differential metabolite profile between the IBS and control groups; a cluster of metabolites was also found to be significantly associated with the amount of defecations. Moreover, the WAS group exhibited a decreased alpha diversity of the microbial population as compared to the control group. However, the characteristics of gut microbiota could not differentiate the IBS group from the control group. Correlation of the metabolite level with the number of microbial genera showed no significant association between the control and IBS groups. This study provides a global perspective on metabolomics and microbiota profiling in WAS-induced IBS model and a theoretical basis for research on the pathophysiology of IBS.

## Introduction

Irritable bowel syndrome (IBS) is a common gastrointestinal dysfunctional disease with a prevalence of 8–20% in many countries, and the number of IBS cases is 1.5- to 2-fold more in women than in men(Lovell and Ford, 2012a,b). IBS is characterized by multiple symptoms, including chronic and recurrent abdominal pain/discomfort and altered bowel habits (Mayer, 2008; Ford et al., 2017). In particular, patients with IBS appear to have a high level of psychosocial stress and a lower quality of life and work productivity (Cashman et al., 2016). According to the Rome IV criteria, IBS can be clinically classified into four subgroups: constipation (IBS-C), diarrhea (IBS-D), mixed (both constipation and diarrhea) IBS (IBS-M), and neither occurring (IBS-U) (Guilera et al., 2005; Oswiecimska et al., 2017).

The underlying causes of IBS are largely unknown and are thought to be heterogeneous. Research on IBS has traditionally focused on genetic predisposition, altered gastrointestinal motility, and increased gut sensitivity (Rogers et al., 1989; Lucak et al., 2017). Currently, increasing evidence indicates that patients with IBS show an upregulation in neural processing between the gut and the brain, which is termed the “brain–gut axis” (Ohman and Simren, 2010; Mayer and Tillisch, 2011). Dysregulated inflammation and immune function are also thought to contribute to the pathophysiology of IBS (Ohman and Simren, 2010). Recently, a limited number of studies implicated the emerging role of microbiome and metabolites as potential biomarkers and treatment targets in IBS (Shankar et al., 2015; Keshteli et al., 2019).

Human gastrointestinal tract is a complex environment that encompasses large amounts of microorganisms, diverse metabolites, and the interplay between microbes and the host (Yamashiro, 2017). The complex metabolic interactions between microbes and host tissues was proved to be critical to the development of various gut disorders such as IBS and ulcerative colitis (UC) (Ghaisas et al., 2016; Lopetuso et al., 2018). It is estimated that there are 10^12^ microorganisms in the colon, including more than 15,000 different bacterial strains (Sartor, 2008). By anaerobic fermentation, gut microbes can degrade large amounts of carbohydrates from diet to different metabolites such as pyruvate, formate, lactate, succinate, bacteriocins, neurotransmitters, and short-chain fatty acids (SCFAs) (Karasov and Carey, 2009; Cremon et al., 2018). SCFAs have been proven to contribute to the interactions between the host and microbiota (Guilloteau et al., 2010). Furthermore, Gargari et al. reported that SCFAs can be used to characterize the IBS subtypes and represent a therapeutic target for IBS (Gargari et al., 2018). Therefore, it is important to investigate gut metabolites and microbiota alterations in IBS, and the integrated bioinformatic analysis is a useful method for this investigation.

Chronic psychological stress plays an important role in developing and exacerbating the symptoms of functional bowel disorders (Ford et al., 2017). Water avoidance stress (WAS) was widely used to induce an IBS-like symptoms in rats, which is a well-established animal model of IBS (Bonaz and Tache, 1994; Moloney et al., 2015). In addition, the microbiome of rats is more similar to that of humans (Wos-Oxley et al., 2012). Therefore, fecal samples from WAS rats, which were established in a previous study (Zhu et al., 2018), were used in the present investigation to clarify the plausible alteration of microbiota and metabolites in the development of IBS.

In this investigation, by using fecal samples from the IBS and control rats, we carried out the following two experiments: metabolite or microbe profiling analysis and correlative microbe–metabolite analysis. This study aimed to investigate whether fecal metabolite and microbiota profiles can be identified as a novel pathophysiological target for IBS.

## Materials and Methods

### Animal Experiment and Sample Collection

Ten male rats (age: 8 weeks, weight: 180 g) were randomized into the control group and the IBS group. Five rats in the IBS group were repeatedly exposed to WAS and five rats in the control group were treated in the same way for 10 days but in a cage without water stress as described in the previous study (Zhu et al., 2018). Briefly, the procedure results in increased bowel movement, including the number of both dry and loose stools, in the IBS group as compared to that in the control group (Zhu et al., 2018). Fecal samples assessed in this study were collected on the last day after 10 consecutive days of WAS stimulation. Ten fecal samples were then immediately stored at −80°C until further processing. Each sample was divided into two parts for metabolite and microbiota analyses.

### Sample Preparation and Fecal Metabolomics Profiling

Fecal samples from each rat were stored at −80°C before the process of quantification. Sample preparation was performed using a test kit—MicrobioMET (Metabo-Profile, Shanghai, P.R. China). Briefly, samples were thawed on an ice bath and weighed 50 mg for preparation. The samples were then homogenized with 1 M NaOH (300 μL) solution and centrifuged at 13,500 rpm at 4°C for 20 min. The supernatant (200 μL) of each sample was transferred into an autosampler vial. The residue was further extracted with 200 μL methanol and centrifuged again under the same conditions. The resultant supernatant was combined with the first one in the autosampler vial. The autosampler vial was then capped, and the extracts were subjected to automated sample derivatization using the multipurpose sampler MPS2 (Gerstel, Muehlheim, Germany). After sample preparation, microbial metabolite panels were detected and quantitated with a gas chromatography coupled to time-of-flight mass spectrometry (GC-TOFMS) system (Pegasus HT, Leco Corp, USA). The type of capillary column used for gas chromatography is Rxi-5MS (30 m × 250 μm I.D 0.25 μm film thickness). The total mass of the metabolites was determined by the metabolite diversity analysis.

### 16S rRNA Gene Sequencing

By using a PowerSoil® DNA Isolation Kit (MO BIO Laboratories, Carlsbad, CA), DNA was extracted from fecal samples according to the manufacturer's instructions. The extracted DNA was quantified using the Nanodrop spectrophotometer (Thermo Fisher Scientific, Massachusetts, USA) and stored at −80°C for further analysis. The V4 regions of the bacterial 16S rRNA gene were amplified by polymerase chain reaction (PCR) using universal primers (U515: 5′-GTGCCAGCMGCCGCGGTAA-3′, E786: 5′- GGACTACHVGGGTWTCTAAT-3′). Each amplicon was then purified and barcoded. The samples were finally pooled to construct the sequencing library and sequenced using an Illumina Miseq (Illumina, San Diego, CA) to generate pair-ended 150 × 150 reads. The raw data of sequencing were then spliced and filtered to obtain the clean data. Subsequently, operational taxonomic units (OTUs) clustering and species classification were performed.

### Data Analysis and Statistical Tests

The visualization and comparison of the metabolite profile were performed using orthogonal partial least squares discriminant analysis (OPLS-DA) with permutation testing algorithm to detect the metabolic variation between the groups. Non-metric multidimensional scaling (NMDS) analysis was used to investigate the difference in gut microbiota between the samples. Unweighted pair-group method with arithmetic mean (UPGMA) analysis was performed to construct a heat map profile of metabolites or microbiota. Univariate statistical analysis was used to perform inter-group comparisons.

An R package named weighted gene co-expression network analysis (WGCNA) was applied to microbiological and metabolomic data analysis. An adjacency matrix was obtained by symbolizing the correlation matrix and the power determined by the scale-free topology. The topological overlap matrix was calculated, and hierarchical clustering analysis was performed. Modules with a correlation coefficient >0.7 were combined into one module. The first eigenvector of the module was used to represent each module. To determine the membership of each gene in the module, the average connectivity of the genes in the module was calculated.

Spearman correlation analysis was used to evaluate the correlation between microbes, metabolites, and pathophysiological features. *P* < 0.05 were considered as significant. A correlation network plot was generated, and correlation magnitudes >0.6 (strong co-abundance relationships) and < -0.6 (strong co-exclusion relationships) were plotted. Visualization of the network was performed by Cystoscope v3.2.2.

## Results

### The Gut Metabolites in the IBS Group Were Significantly Different From Those in the Control Group

**Figure 1:**
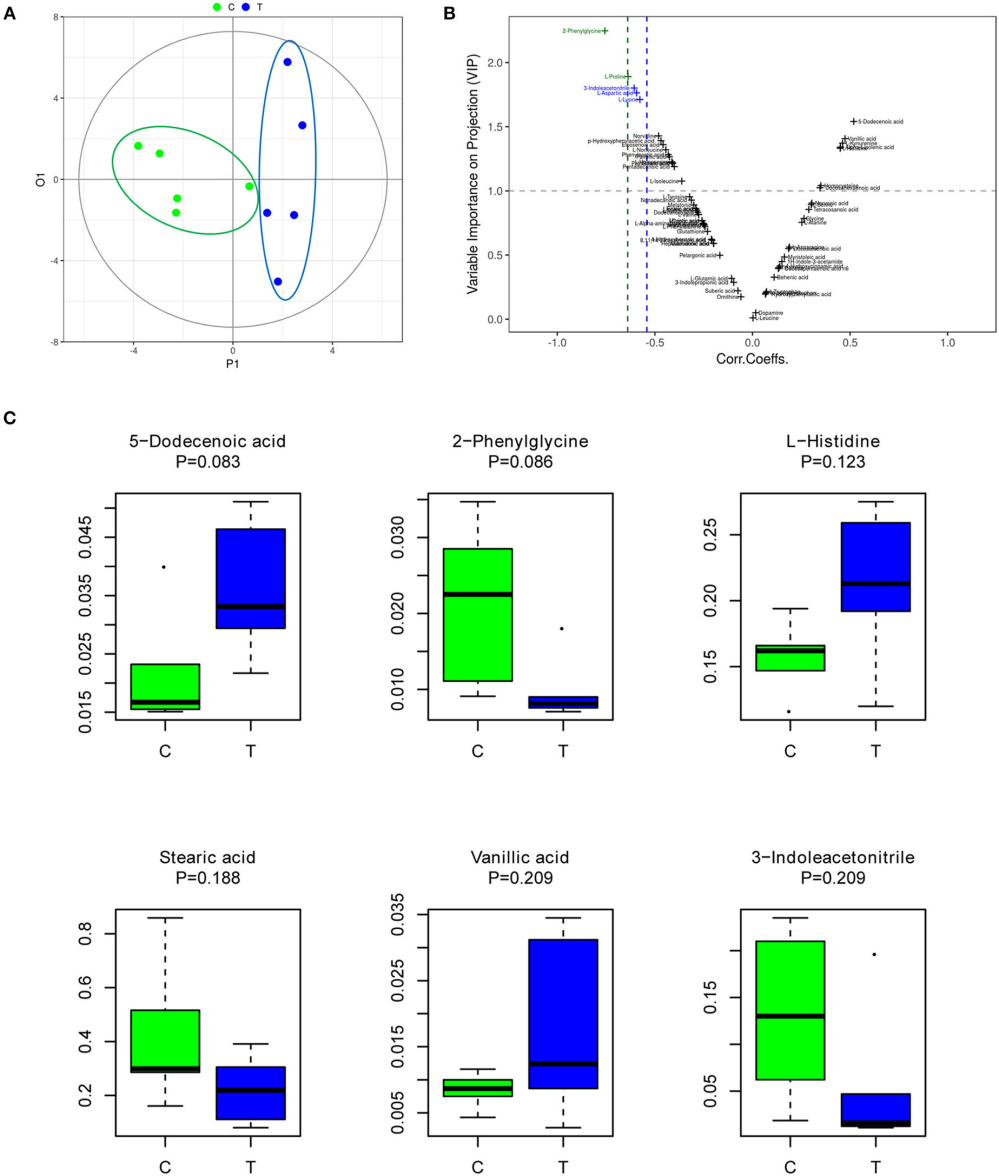
The gut metabolites of IBS rats were significantly different from those of the control group. **(A)** Plot of OPLS-DA scores of the control (green) and IBS (blue) groups. **(B)** Variable importance in projection (VIP) score plot for metabolite features identified by OPLS-DA. Metabolites with VIP score >1.0 were considered to be significantly different. **(C)** The abundance of representative metabolites in the IBS group compared to that in the control group.

OPLS-DA is an efficient multivariate statistical method for data analysis of metabolite profiling (Khan et al., 2008; Huang et al., 2010). OPLS-DA revealed that the gut metabolites in the control group were significantly different from those in the IBS group. The plot of OPLS-DA scores is presented in Figure 1A. Each spatial point in the K-dimensional space represents a single sample, and the samples were color-coded according to the grouping information. P1 represents the variance components of the X-matrix, and O1 represents the Y-matrix, and the results in the figure are shown in the P1 dimension. The IBS group (blue) and the control group (green) were clearly separated. This result provides a theoretical basis for further analysis of differential expression of metabolites. Therefore, we continued to identify which metabolites were more likely to have an impact on predicting IBS by computing variable importance in projection (VIP) scores of each metabolite (Figure 1B). According to the VIP score plot, metabolites with VIP score >1.0 were further analyzed. Figure 1C shows the top 6 metabolites in the rankings, according to their importance for separating the two groups (Figure 1C). The six metabolites included 5-dodecenoic acid, 2-phenylglycine, L-histidine, stearic acid, vanillic acid, and 3-indoleacetonitrile. Among the metabolites, 5-dodecenoic acid, L-histidine, and vanillic acid tended to be upregulated, while 2-phenylglycine, stearic acid and 3-indoleacetonitrile tended to be downregulated. 2-Phenylglycine and L-histidine are amino acids; 5-dodecenoic acid and stearic acid are long-chain fatty acids; vanillic acid is a monohydroxybenzoic acid; and 3-indoleacetonitrile is a phytoalexin.

### Cluster Analysis of Gut Differential Abundant Metabolites in the IBS Group

**Figure 2:**
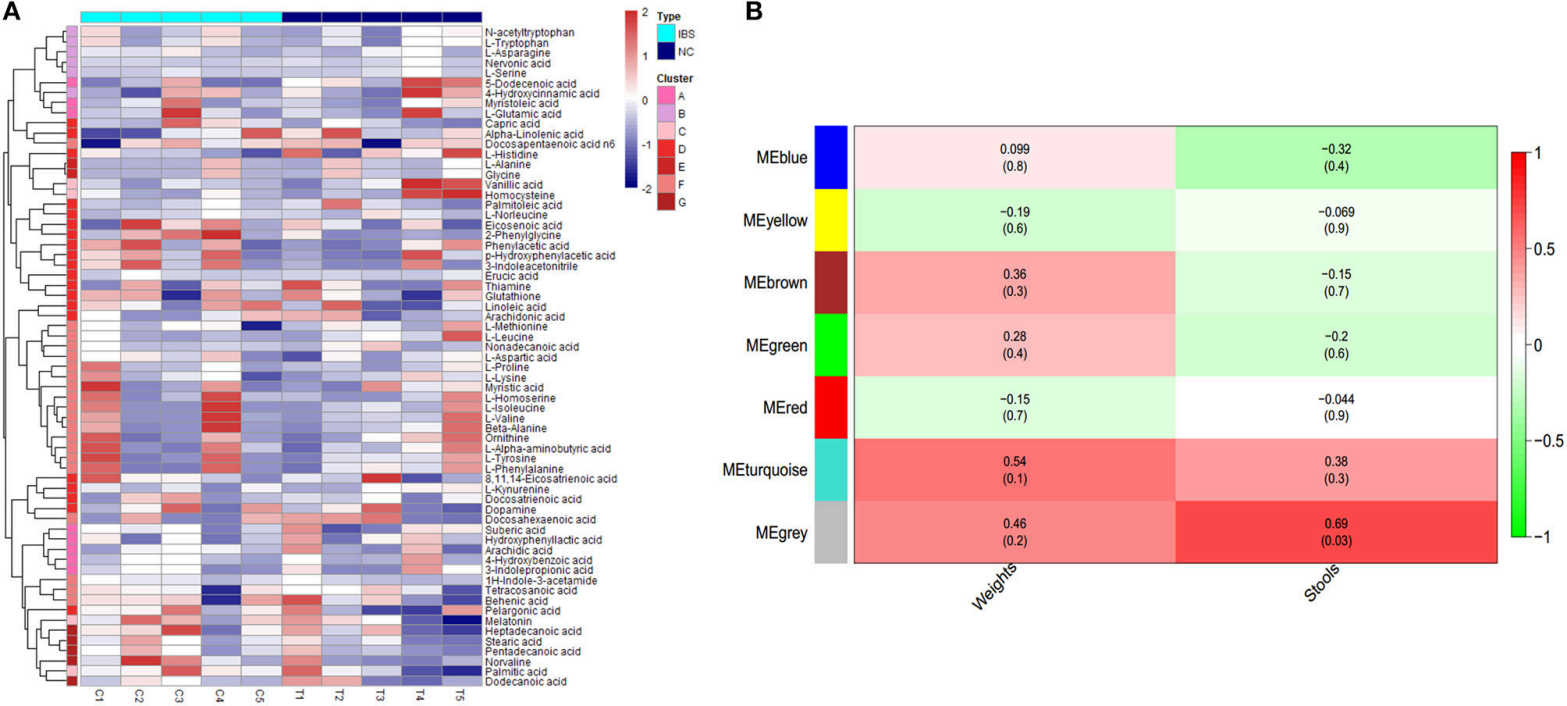
Cluster analysis of differential metabolites in IBS rats. **(A)** The heatmap shows the abundance of metabolites in different clusters from each sample. **(B)** The heatmap shows the correlations between metabolites and different phenotypes of rats (weight and stools).

To identify the different clusters of metabolites in the control and IBS groups, UPGMA analysis was performed to construct a heat map profile (Figure 2A). The left column with 7 different colors shows that metabolites were clustered into 7 groups. In addition, the possible inherent association between differential metabolites and clinical trait was investigated with a constructed correlation matrix (Figure 2B). In this figure, different colors on the left side represent the different clusters of metabolites, and the right gradient color band represents the degree of correlation. The metabolites in the cluster of MEgrey were significantly associated with the amount of defecation in IBS rats (*R* = 0.68, *P* = 0.03).

### Quantitative Profiling of Gut Microbiota in IBS Rats

**Figure 3:**
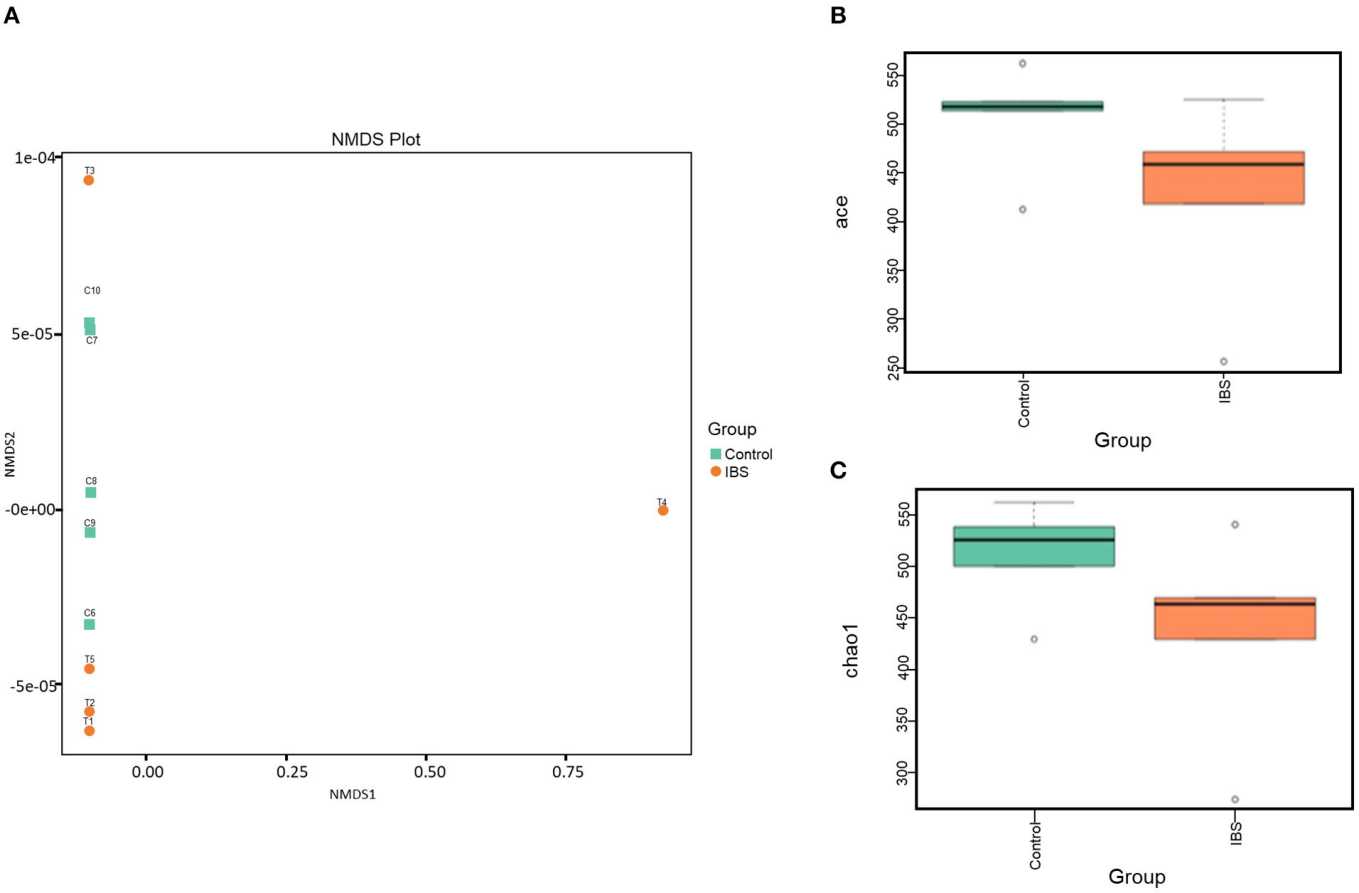
The gut microbial analysis of IBS rats. **(A)** Non-metric multidimensional scaling (NMDS) plot of the control (orange) and the IBS (green) group. **(B,C)**. Alpha diversity of ACE **(B)** and Chao1**(C)** indices of the control and IBS groups.

16S rRNA gene sequencing was performed to estimate fecal microbiome diversity in IBS rats. The total number of effective tags and OTUs in each fecal sample is shown in Supplementary Figure 1A. Further analysis of OTUs indicated that the control and IBS rats had similar abundance of different bacterial families at each taxonomic level (Supplementary Figure 1B). In addition, NMDS analysis was used to investigate the difference in gut microbiota between the samples. According to the distance between the sites, the NMDS results (based on the OTUs) indicated that partial IBS sites were clustered together and these IBS sites was significantly different from the control sites (Figure 3A). It has been reported that a change in microbial diversity (alpha diversity) is involved in gut disorders. Therefore, the alpha diversity index for fecal microbiomes was compared between the control and IBS rats. Both abundance-based coverage estimator (ACE) and Chao1 indices indicated that the level of gut microbial diversity in the IBS group was significantly lower than that in the control group (Figures 3B,C).

### Cluster Analysis of Gut Differential Abundant Microbiota in IBS Rats

**Figure 4:**
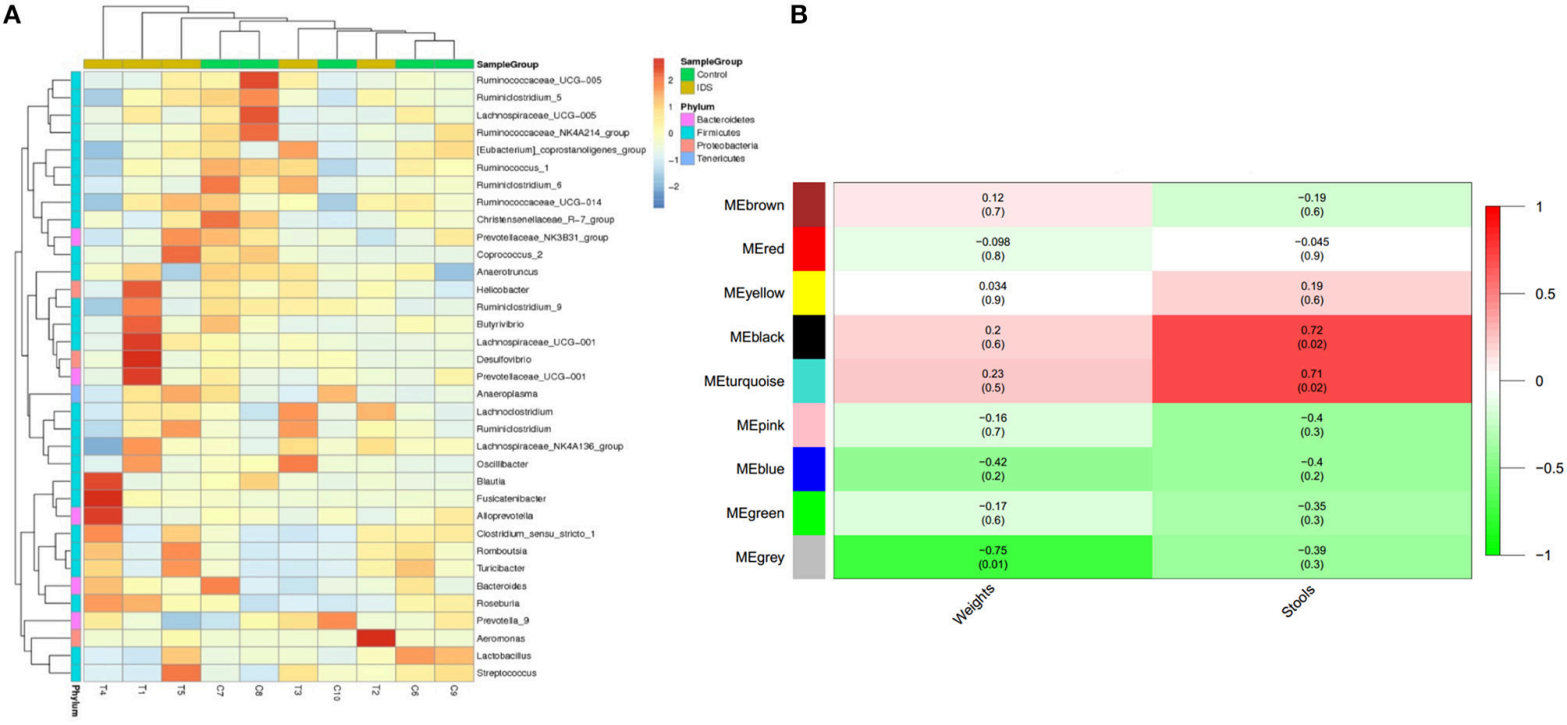
Cluster analysis of gut microbes in IBS rats. **(A)** The heatmap shows the abundance of microbes in different clusters from each sample. **(B)** The heatmap shows the correlations between microbes and different phenotypes of IBS rats (weight and stools).

To investigate the difference in microbial abundance between the control and IBS groups, a heat map profile was constructed (Figure 4A). The left column with 4 different colors in the heat map indicates that the differentially abundant microbes were clustered into 4 groups. The clustering indicated that the IBS and control groups had similar characteristics of the gut microbiota; this might be due to the limited sample size and various factors that affect gut flora. In addition, the possible inherent association between differential microbes and clinical trait was investigated with a constructed correlation matrix (Figure 4B). In this figure, different colors on the left side represent the differentially abundant microbes, and the right gradient color band indicates the degree of correlation. The black and turquoise clusters were significantly associated with the amount of defecation in IBS rats.

### Association Analysis of Differentially Abundant Microbes and Metabolites

**Figure 5:**
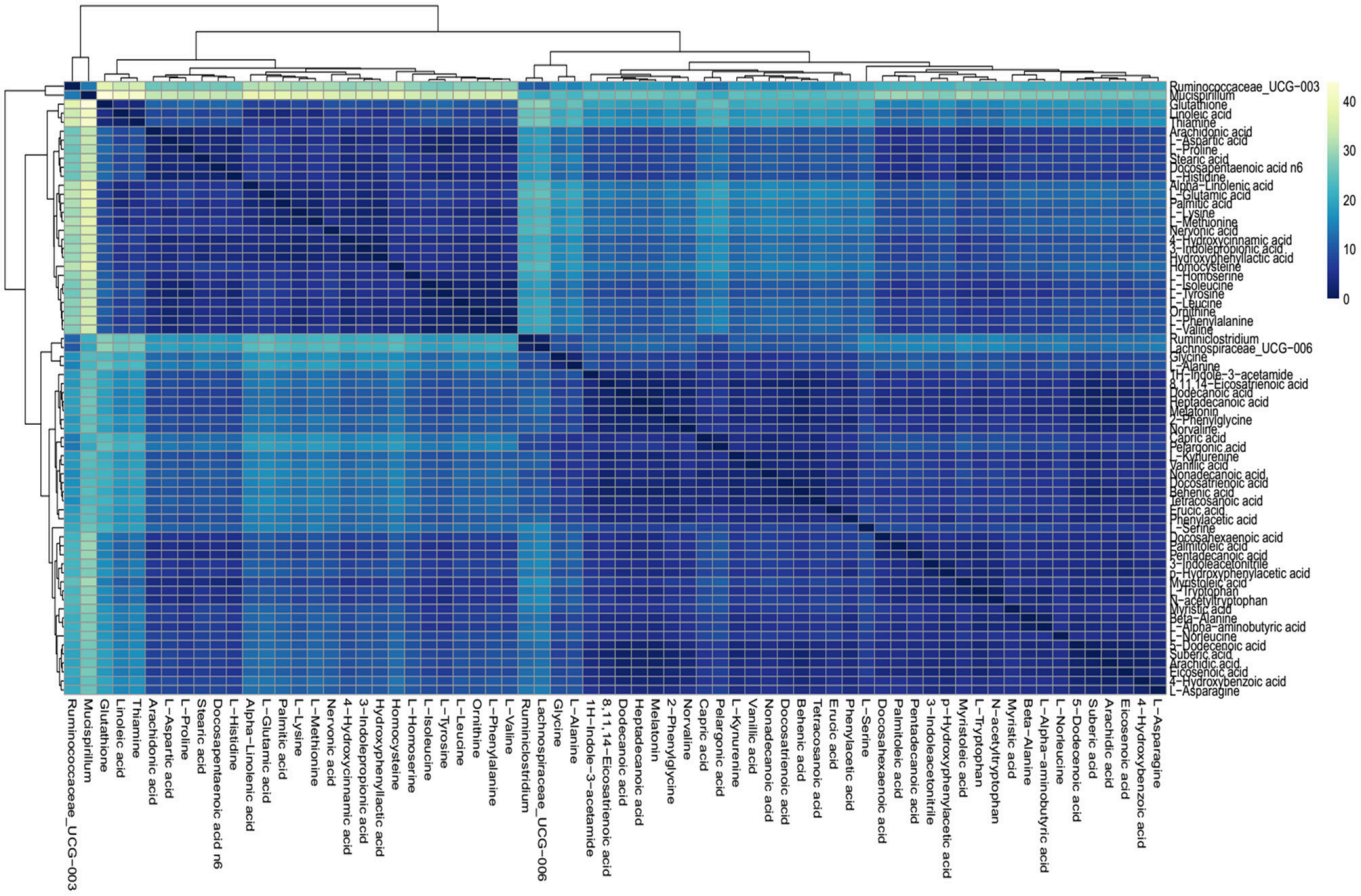
Association analysis of differential gut microbes and metabolites. Correlation matrices between microbial genus abundances and fecal metabolites. The right gradient color band indicates the *P*-value of correlation. The results of the association of differentially abundant microbes and metabolites showed that the metabolites were significantly correlated with each other and the microbiota was also significantly correlated with each other, but the correlation between the microbes and metabolites was weak.

On the basis of the discovery of differential profiling of the gut metabolites and microbes between the IBS group and the control group, the possible association between metabolites and microbial genus abundance was further investigated by computing the correlation matrix (Figure 5). The results showed that the differential metabolites were significantly correlated with each other. However, the association between intestinal metabolites and microbes was weak, suggesting that the changes in gut microbes and metabolites during the pathogenesis of IBS may be two independent biological processes.

## Discussion

The pathophysiology of IBS is largely unknown and heterogeneous. Many factors can contribute to the development of IBS, for example “brain–gut axis,” immune regulation disorder, altered gastrointestinal motility, risk gene mutations, and so on (Holtmann et al., 2016; Defrees and Bailey, 2017). The present study was carried out to obtain quantitative metabolite and microbe signatures of fecal samples from WAS-induced IBS rats and control rats; this investigation also aimed to evaluate possible microbe–metabolite associations in the intestinal micro-environment. To our knowledge, this is the first report that analyzed the networks of gut microbe–metabolite associations in WAS-induced IBS rats.

In this study, we demonstrated a significantly differential metabolite profile between the IBS and control groups, suggesting that the changes in gut metabolites might play an important role in the development of IBS and may be potential diagnostic markers for IBS. However, no significantly different specific metabolite was identified in the IBS group as compared to that in the control group. The possible reasons for this may be the small sample size and the individual diversity within a group. However, the VIP score of six metabolites were above 1.0, indicating their significant role in the differentiation between IBS and control rats. Among the metabolites, 3 metabolites tended to be upregulated (5-dodecenoic acid, L-histidine, and vanillic acid) and 3 metabolites tended to be downregulated (2-phenylglycine, stearic acid, and 3-indoleacetonitrile).

Histidine is a semi-essential amino acid required for growth and tissue repair in humans. Consistent with our findings, Keshteli et al. also reported that patients with IBS have higher urinary histidine level, which is correlated with the severity of IBS (Keshteli et al., 2019). Interestingly, our study found that histidine is included in the cluster of metabolites that is associated with the amount of defecation in IBS rats; this finding suggests the role of histidine in the severity of IBS. Vanillic acid is a plant metabolite that is mainly produced by catechol metabolism (Pietta et al., 1998). Choi et al reported that pretreatment with a hot water extract of the branches of *Hovenia dulcis* (WEHD) significantly increased the frequency and weight of stools in rats. Interestingly, vanillic acids were identified as an effective component in WEHD and exhibited spasmogenic activity (Choi et al., 2018). Therefore, our results suggest that increased vanillic acid level may be involved in the development of IBS with diarrhea (IBS-D). 2-Phenylglycine is a metabolite found in normal human urine and plasma (Miettinen, 1962; McEwen, 1965). In the present study, we found for the first time that 2-phenylglycine is a metabolite in fecal sample in IBS rats. Stearic acid is a straight-chain saturated fatty acid that is identified as a human metabolite and a component of many lipids. Recently, Ye et al. also found a decreased level of stearic acid in the fecal sample of mice fed with methionine-choline-deficient (MCD) diet. Moreover, they reported that MCD diet results in gradual intestinal barrier (Ye et al., 2018). Combining the results of Ye et al with our research on IBS rats, we speculated that decreased stearic acid level may contribute to the increased intestinal permeability in IBS. In addition, the metabolites of cluster MEgrey were found to be significantly associated with the amount of defecation, thus suggesting a possible inherent association between metabolites and IBS clinical traits, which is consistent with other studies (Shankar et al., 2015; Keshteli et al., 2019).

We found that no significant difference in microbiota between the IBS group and the control group. However, Fourie et al found significant changes in colonic mucosa-adherent microbial taxa and clades in WAS rat model and reported a trend of increased microbial diversity and richness (Fourie et al., 2017). Compared with their study, the sample analyzed in our present study is feces, which is more convenient for clinical application in the future. We also found that the diversity of gut flora in the IBS group was significantly lower than that in the control group; this finding is consistent with most studies conducted using human datasets (Le Chatelier et al., 2013; Pittayanon et al., 2019). The results suggested that patients with IBS might lose the diversity of intestinal flora, which may play an important role in the pathogenesis of IBS. Nevertheless, no significantly differential microbe was identified in this study; this might be due to the limited sample size and various factors that affect the gut flora, leading to the individual difference within each group. Thus, it is important to increase the sample size in the future study.

The association study of differentially abundant microbes and metabolites showed that the metabolites were significantly correlated with each other and the microbiota was also significantly correlated with each other. However, the correlation between the microbes and metabolites was weak, suggesting that the changes in gut microbiota and metabolites during the pathogenesis of IBS may be two relatively independent biological processes. The lack of strong metabolite–microbe interactions in IBS rats might be due to the broad etiology involved in IBS development (Ghaisas et al., 2016). Different pathophysiological conditions that lead to IBS symptoms may result in unique shifts of microbiota community structure and/or functional capacity in different individuals (Watanabe et al., 2012). However, the small sample size may have led to the insignificantly different results during the analysis. Thus, the results of the present study need to be confirmed by further investigation with a large sample size.

The present study has the following limitations. The study analyzed relatively limited sample size, which may limit the accuracy and reproducibility of metabolite analysis in IBS and control rats. However, the results of the present study are partially consistent with those of previous studies. Thus, our study may be an interesting pilot study for a more extensive research project in the future.

Taken together, this study indicates that it might be worthwhile to investigate the members of differential abundant metabolites and to elucidate their role both in the metabolic processes in the gut and their potential interplay with the intestinal permeability and nervous systems. Further study of these metabolites in larger clinical trial studies will help to determine the prognostic value of these metabolic features and their potential role in pathogenesis and future treatments.

## Ethics Statement

The animal protocol was approved by the Ethics Committee for Animal Study in Beijing Friendship Hospital. The investigation conforms with the Guide for the Regulation to the Care and Use of Experimental Animals of the Beijing Council on Animal Care (1996).

## Author Contributions

SheZ and LM designed the experiments. SL, LC, HL, YY, and ZZ performed the experiments. SL, CS, LM, GZ, and YC analyzed the data. SL and CS wrote the manuscript. ShuZ revised the manuscript. All authors have approved the final version of the manuscript and agree to be accountable for all aspects of the work, ensuring that questions related to the accuracy or integrity of any part of the work are appropriately investigated and resolved.

### Conflict of Interest Statement

The authors declare that the research was conducted in the absence of any commercial or financial relationships that could be construed as a potential conflict of interest.

## Supplementary Material

Supplemental Figure 1 **(A)** The total number of effective tags and operational taxonomic units (OTUs) in each fecal sample; **(B)** Analysis of OTUs indicated that control and IBS rats had similar abundance of different bacterial families at each taxonomic level.
