# Supplementary figures and images for: Multi-omics Analysis of Gut Microbiota and Metabolites in Rats With Irritable Bowel Syndrome
Source: Front Cell Infect Microbiol. 2019 May 29;9:178. doi: 10.3389/fcimb.2019.00178 (PMC6549239; doi:10.3389/fcimb.2019.00178)

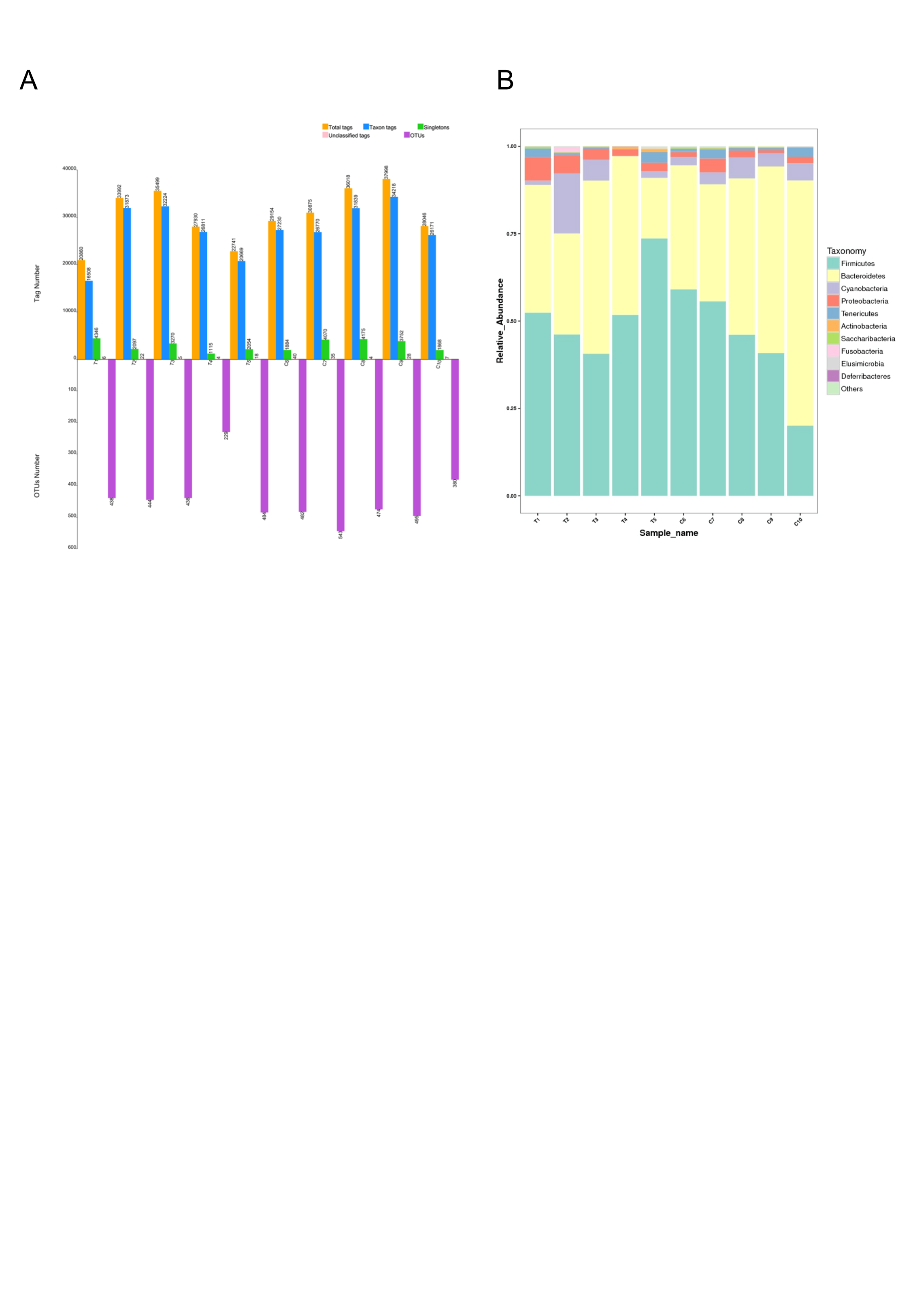

Supplement: Supplemental Figure 1 — (A) The total number of effective tags and operational taxonomic units (OTUs) in each fecal sample; (B) Analysis of OTUs indicated that control and IBS rats had similar abundance of different bacterial families at each taxonomic level. [file Image_1.TIF]
